# Sectoral Productivity Growth, COVID-19 Shocks, and Infrastructure

**DOI:** 10.1007/s41885-021-00098-z

**Published:** 2022-01-21

**Authors:** Hildegart Ahumada, Eduardo Cavallo, Santos Espina-Mairal, Fernando Navajas

**Affiliations:** 1 Universidad Torcuato di Tella (UTDT), Buenos Aires, Argentina; 2 Inter-American Development Bank (IDB), Washington, USA; 3 Fundación FIEL, Buenos Aires, Argentina; 4 Universidad de Buenos Aires, Buenos Aires, Argentina

**Keywords:** COVID-19, Sector shocks, Productivity, Infrastructure, O47, C51

## Abstract

This paper examines sectoral productivity shocks of the COVID-19 pandemic, their aggregate impact, and the possible compensatory effects of improving productivity in infrastructure-related sectors. We employ the KLEMS annual dataset for a group of OECD and Latin America and the Caribbean countries, complemented with high-frequency data for 2020. First, we estimate a panel vector autoregression of growth rates in sector level labor productivity to specify the nature and size of sectoral shocks using the historical data. We then run impulse-response simulations of one standard deviation shocks in the sectors that were most affected by COVID-19. We estimate that the pandemic cut economy-wide labor productivity by 4.9% in Latin America, and by 3.5% for the entire sample. Finally, by modeling the long-run relationship between productivity shocks in the sectors most affected by COVID-19, we find that large productivity improvements in infrastructure—equivalent to at least three times the historical rates of productivity gains—may be needed to fully compensate for the negative productivity losses traceable to COVID-19.

**Supplementary Information:**

The online version contains supplementary material available at 10.1007/s41885-021-00098-z.

## Introduction

The COVID-19 pandemic is one the largest and most intricate economic disruptions of modern history. While pandemics or health crises of various sorts have had economic consequences before (Bloom et al. 2020; Dieppe 2020), previous events were less globalized because they were confined to certain regions and because they occurred in economies less marked by the fluid interactions that characterize modern economies, both advanced and emerging.

The new reality is societies with an ever-growing degree of social interaction and high mobility at the local, regional, and global levels (see Nakamura and Managi 2020). In such a context, the speed of transmission of a disease, captured by the coefficients of epidemiological models, has been fast and variable across regions and economic sectors. The combination of government intervention and social response to COVID-19 (see for example, Katafuchi et al. 2020) has led to short- and long-term uncertainties (the timing of the introduction of effective vaccines being one of them), multiple phases (Moore et al. 2020; Ahumada et al. 2020; Baqaee et al. 2020), and an ongoing adaptation of economic activity—all resulting in a macroeconomic cycle different from previous crises.

This view is implicit in recent assessments of the economic effects of COVID-19 (IMF 2020a, b), since forecasts depend on the interaction between government policies and social responses, which in turn lead to a rather cautious view of the speed of recovery, with possibly marked differences between advanced and emerging economies. The explanations of the slow expected recovery go beyond the effects of fiscal and monetary policies to the interaction between health and economic outcomes under uncertainty and a “drag” caused by a reallocation shock that the economy needs to process (Barrero et al. 2020; Barrero and Bloom 2020). This “slow exit” hypothesis rests on evidence that high and sustained uncertainty due to COVID-19 fuels expectations of downside risks, which then exacerbate the recession, slow the recovery, and reduce the effectiveness of policy interventions, including current vaccination programs.

While attention has been focused on the interactions among the spread of the pandemic, the effects of interventions, and the effects of behavioral responses on aggregate economic activity and employment (IMF 2020a), there is less evidence on the nature of the economic effects and the process of transmission of supply and demand shocks in given sectors after COVID-19. Several papers have studied the nature of the impacts on multi-sector economies. Brinca et al. (2020a, b) follow a decomposition proposed by Baumeister and Hamilton (2015) and use a SVAR estimation to classify, using U.S. labor data, supply and demand shocks across sectors. They obtain results that show that two-thirds of initial shocks were supply-side shocks concentrated in certain subsectors of domestic services (hospitality, such as hotels, restaurants, etc.), construction, and manufacturing. By contrast, infrastructure-related sectors, such as utilities and transport, suffered less, while other sectors, including information and financial services, fared relatively well.

Theory-based models like that of Baqaee and Farhi (2020) study the effects of the COVID-19 crisis in a disaggregated Keynesian model with multiple sectors, finding that negative supply shocks are stagflationary, and negative demand shocks are deflationary. Guerrieri et al. (2020) show that in a model of multiple sectors and incomplete markets, and under certain assumptions, supply shocks can have effects that resemble demand shocks. Other theoretical models incorporate aspects of epidemiology into standard macroeconomic models; here, epidemics generate reductions in economic activity that are captured as negative supply and demand shocks (Eichenbaum et al. 2020). Some computable multisector models were implemented early on in the pandemic to simulate the global effects of lockdowns using input–output data (Mandel and Veetil 2020). Finally, going beyond aggregate intersectoral models, papers that study allocative shocks after COVID-19 have used firm data based on expected sales and employment to look at intrasectoral reallocations (Barrero et al. 2020). According to their vision, much of the allocative effect occurs within parts of the services sector, instead of across sectors.

In the case of the countries of Latin America and the Caribbean (LAC), evidence is scant concerning the magnitude and nature of shocks following the COVID-19 pandemic. The lacunae extend to the differential nature of contraction in activity and employment, its sectoral decomposition, and, finally, the prospects for recovery. Available evidence (IMF 2020b) shows an employment contraction more severe than in advanced economies, associated with the stylized fact that unemployment is concentrated in hard-hit sectors such as services and construction and where small-size firms, informality, or soft contracting without job protection is pervasive. This evidence shows that the adjustment falls asymmetrically on sectors and firms that rely more heavily on informal labor contracts and have a relatively large (negative) productivity gap, low capital intensity, and low productivity.^1^ Finally, while there is limited evidence (IMF 2020b) on the role of informality in adjustments to the COVID-19 crisis, some stylized facts on extensive and intensive margins of mobility across income deciles and across urban populations (Aromi et al. 2020) show that in the first decile of the income distribution, where informality approaches 90% in LAC economies, a faster recovery of mobility is found.

This paper attempts to fill the gap in our knowledge about the sector-level effects of the COVID-19 pandemic, with a focus on LAC. The unprecedented global scope of the pandemic complicates the task of benchmarking the pandemic to previous crises. The global crisis of 2008–2009 was characterized chiefly by productivity losses in the manufacturing sector owing to an interplay of international trade and financial shocks. The effects of COVID-19, by contrast, seem to be located in service subsectors, where demand and/or supply were constrained because of restrictions and social behavior. The losses in these subsectors may have had direct effects on the economy and indirect ones through their effect on other sectors—and in a way that may have lasting consequences on the productivity path of the economy.

While the type and size of shocks may be different, their transmission across sectors can have lasting consequences, as explained, for example, in the sudden-stop literature (see Calvo et al. 2006) and in the study of total factor productivity (TFP) in LAC (Daude and Fernández-Arias 2010). Cross-sectoral transmission is also supported by Cavallo and Powell (2021), who use the KLEMS dataset to study the effect of macroeconomic crises on TFP in LAC, separating sectors by their capital intensity. In fact, capital-intensive sectors appear to have suffered lower output losses in the wake of COVID-19 (Brinca et al. 2020a, b). It is likely that they will adjust more easily to a new normal because of their high productivity. Among them, infrastructure-related sectors such as utilities, transport, and logistics may gain in productivity, exerting long-run effects on aggregate productivity growth. Thus, one might posit that economies were shaped by the pandemic as a function not only of the *magnitude* of the shocks they suffered, but also of their sectoral distribution. The flip side is that productivity growth in infrastructure may help to compensate for COVID-19 shocks by providing more efficient services and allowing demand to manifest itself more easily in the more affected sectors—thereby facilitating the economy’s adaptation to the new normal.

Testing this hypothesis requires an empirical approximation of the relationship between sectoral productivity growth, COVID shocks, and infrastructure. With that in mind, we build on the literature on productivity growth, macroeconomic shocks and the interplay of infrastructure and growth.

The global productivity slowdown that took place after the great recession of 2008–2009 posed challenges for advanced and emerging economies alike—and these have increased with COVID-19 (Dieppe 2020). Against this background, infrastructure investment can be a conduit to increasing productivity growth (Ahumada and Navajas 2019). Much of the literature on the effects of infrastructure since Aschauer (1989) view it as capital additions (public and private) that stimulate aggregate productivity and economic growth. More recently Ramey (2020) offered a solid elaboration on the interplay between infrastructure and aggregate output, separating short- and long-run effects. With respect to empirical research, the growth-infrastructure relationship has been profusely tested at the level of the economy, with results varying according to the types and forms of physical infrastructure (see, for example, Calderón et al. 2015; Calderón and Servén 2016; Estache and Garsous 2012; Égert et al. 2009), but all point to the relevance of infrastructure for long-term growth.^2^

Evidence of the insufficiency of infrastructure investment in many emerging economies, including some in LAC, has come from an approach that measures investment gaps (see for example Fay et al. 2017; Dieppe 2020; Borensztein et al. 2014). However, the estimated gaps may not be reliable enough to guide priorities in a growth strategy based on what types of investment contribute most to raising per capita income (Izquierdo et al. 2016), or in a broader sustainable strategy (Rozenberg and Fay 2019).

Another approach points to the need to focus on the “software” side of infrastructure provision (Cavallo et al. 2020). This approach highlights the need to focus on infrastructure as a service, particularly in regions such as LAC that have fiscal constraints and regulatory environments that make it difficult to close investment gaps quickly by increasing capital stocks exclusively. Within this broader vision, Ahumada and Navajas (2019) evaluate the effects that increases in the productivity of infrastructure-related sectors have on other sectors. They do so within a productivity-growth framework in which productivity in infrastructure-related sectors affects productivity growth in other sectors of the economy and thus exerts direct and indirect effects on aggregate productivity growth. They test sectoral effects in 25 countries using data from the Groningen GGDC dataset (Timmer et al. 2007, 2015). Employing an automatic selection procedure (and taking into account exogeneity and cross-dependance), the authors find several cointegrated relationships between the productivity of labor and capital in utilities, transport, and construction and that of several other sectors, from agriculture to services (wholesale, retail, and hospitality). Indirect effects of productivity improvements in infrastructure-related sectors are quantitatively more significant than direct effects, pointing to significant spillovers on other sectors.^3^

This paper uses the growth-accounting KLEMS dataset for a group of eight LAC countries (LAKLEMS 2020; IDB and IVIE 2020; Mas and Benages, 2020; Hofman et al. 2017a, b). Combining this dataset with its sample-compatible counterpart for a group of 16 OECD countries (available for 1995–2015) allows us to study intersectoral transmission of shocks, which we use to calibrate a simulation of the COVID-19 shocks.

Section 2 begins with a description of sectoral TFP shocks apparent in the KLEMS dataset for the years 1995–2015. We move on to gauge the shock from COVID-19 by tracking the changes in monthly sectoral output over 2020 in most of the countries of the KLEMS sample. In Sect. 3, we estimate a panel vector autoregression (PVAR) of sectoral rates of growth in labor productivity to characterize the nature and size of sectoral shocks for the OECD and LAC countries in the KLEMS dataset.

Using the PVAR estimates, we then perform an impulse response simulation for shocks of one standard deviation in sectors such as wholesale, retail, and hospitality services; construction; and manufacturing, which we conjecture to be a good approximation of observed first-round shocks from COVID-19 in 2020. Separating estimates for the whole sample from those for LAC countries, we show that the latter suffered greater shocks. In Sect. 4 we compute direct and indirect effects of the selected shocks to labor productivity. We find that in the aggregate, effects in the three sectors add up to a 4.9% hit to economy-wide labor productivity in LAC and a 3.5% hit in the sample as a whole.

In Sect. 5 we assess how much the productivity of infrastructure-related sectors would have to improve to compensate for the losses ascribable to COVID-19. We proceed by implementing—for the KLEMS dataset running from 1995 to 2015—the framework proposed in Ahumada and Navajas (2019) to estimate long-run relationships between productivity in wholesale, retail, commerce and hospitality, services (the sector most affected sector by COVID-19) shocks, and productivity improvements in infrastructure-related sectors.

Specifically, we carry out an exercise in which we raise the productivity of infrastructure in order to observe the likely effects of such improvements on other sectors, chiefly wholesale, retail, and hospitality services (the subsectors most affected by COVID-19), but also manufacturing and construction. These are the sectors that, according to Brinca et al. (2020a; b) and from the evidence offered by the 2020 data, provide the appropriate characterization, qualitative and quantitative, of the COVID-19 shock. The rest of the sectors play a much more passive role. We then ask what degree of improvement in infrastructure productivity would be sufficient reverse the effect of this shock.

Some policy implications and lines of further research are laid out in Sect. 6.

## Productivity Shocks in Recent Global Crises: 2009 and 2020

The KLEMS dataset used in this paper focuses on the differences between OECD and LAC. Online Appendix A summarizes the dataset, along with the control variables we used in our econometric analysis. We cover a total of 24 countries, 16 of which are OECD countries; the coverage includes 11 European Union (EU) countries and eight LAC countries.^4^ The database covers 20 years (1995–2015) of compatible country data and, although it is not as extensive as the GGDC productivity dataset (Timmer et al. 2015) used in a previous study (Ahumada and Navajas 2019), it is based on a growth-accounting framework compatible across countries.

**Fig. 1:**
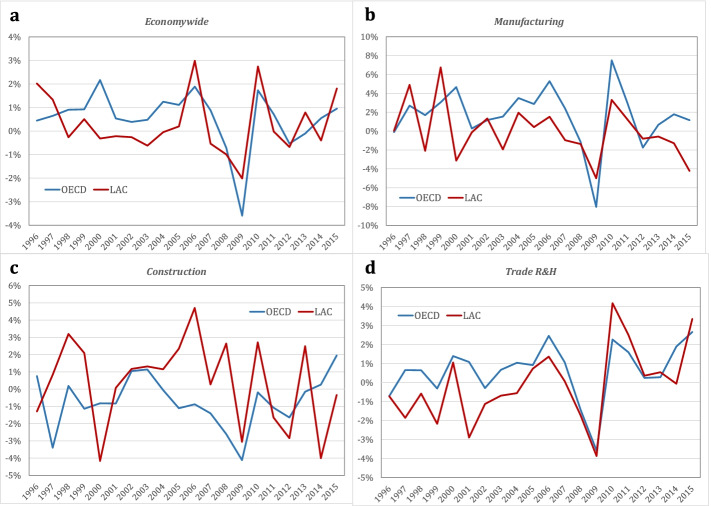
TFP growth in OECD and LAC, 1995–2015, using KLEMS dataset

To learn how changes in the sectors that were particularly affected by the pandemic induced effects in other sectors, we must first understand the magnitude and sectoral distribution of shocks from previous events, particularly the global crisis of 2008–2009. Figure 1 illustrates the annual change in TFP computed in the KLEMS dataset for OECD and LAC, expressed as an unweighted average of TFP changes for countries. Figure 1a is for the economy as a whole; 1b is for manufacturing; 1c, wholesale, retail, and hospitality; and 1d, construction. Table 1 shows all annual TFP rate changes in 2009 for the economy and all sectors and computes ratios between sectoral changes and aggregate changes.

**Table 1:** TFP shocks in OECD and LAC, 2008–09, using KLEMS dataset

Annual change in the TFP Index in 2009				
	OECD	LAC	Sectors ratios to economy-wide	
Economy-wide annual change in TFP	− 3.6%	− 2.0%		

*Sectors TFP annual change*	OECD	LAC	*OECD*	*LAC*
Agriculture	0.2%	− 1.6%	− 0.06	0.82
Mining	− 9.8%	− 8.5%	2.72	4.23
Manufacturing	− 8.0%	− 5.0%	2.23	2.48
Utilities	− 5.4%	− 2.5%	1.52	1.25
Construction	− 4.1%	− 3.1%	1.15	1.52
Domestic trade	− 3.6%	− 3.9%	1.00	1.92
Transport	− 4.4%	0.6%	1.22	− 0.31
Financial services	− 2.2%	− 0.3%	0.62	0.13
Social and public services	− 0.6%	0.9%	0.16	− 0.44

Data show that the TFP shock of the 2008–2009 crisis varied across sectors and regions. In 2009, OECD countries had almost twice the TFP drop as LAC. Manufacturing and mining (with quite different shares in the economy) suffered the largest drops in both regions, but wholesale and retail services and construction (which represent larger shares of employment and value added) suffered relatively more in LAC. Other sectors fared differently; some (agriculture in the OECD and transport and social and public services in LAC) experienced no TFP shock. Thus, LAC did better than the OECD in 2009, but this tended to be in less capital-intensive sectors such as wholesale, retail, and hospitality and in construction, which have large employment shares.^5^

The foregoing evidence helps in characterizing the sectoral distribution of productivity shocks in the 2008–2009 crisis, but it immediately begs the question of the differences with the current COVID-19 crisis, as the KLEMS dataset does not extend to the present. Nor do other productivity datasets, such as the one reported by GGDC. Other studies (such as Brinca et al. 2020a; b), using high-frequency data on U.S. labor statistics, have decomposed supply and demand shocks for the United States and provide a clear picture of supply shocks in hospitality (but not so much in wholesale and retail trade) while manufacturing is among the less-affected sectors. However, extensions of this methodology to other OECD countries or even to LAC countries, which would make it possible to capture differences between the COVID-19 event and the global financial crisis, are not available.

To partially approximate this query, we explore high-frequency data from other sources to capture the magnitude and sectoral distribution of the COVID shock on output in OECD and LAC countries and compare them (using the same source) with the 2008–2009 crisis. Specifically, we collected data on monthly sectoral output indicators that usually constitute a monthly approximation of GDP or activity. Online Appendix B reports sources and links to the dataset constructed for this purpose.

**Fig. 2:**
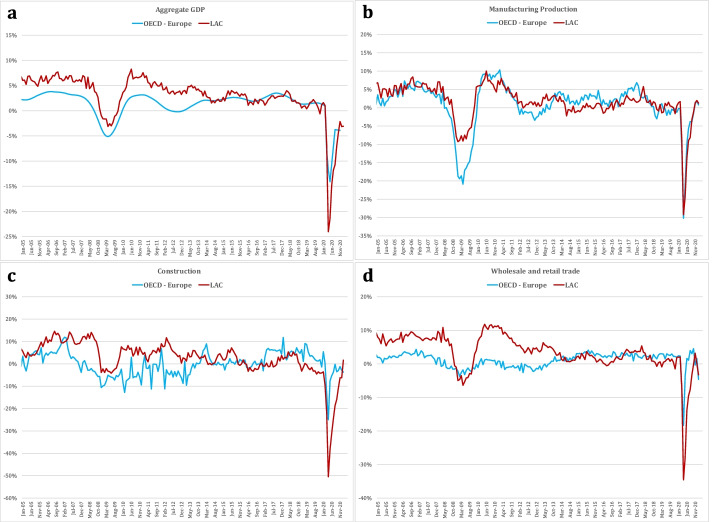
Short-term activity indicators of year-on-year growth rates of GDP, manufacturing, retail trade, and construction in OECD-Europe and LAC, January 2005–December 2020

Figure 2 shows the monthly year-on-year seasonally adjusted unweighted average growth rate of GDP, manufacturing, wholesale and retail trade, and construction for a group of European OECD countries and LAC countries from January 2005 to December 2020. Table 2 compares 2009 and 2020, measuring the year-on-year drop of the worst month of the crisis for the economy, and the ratio of each sector’s growth performance to economy-wide growth. These are measured for different months, as the minimum was reached in a different month in each sector. Table 2 also includes the year-on-year growth rate for the last available month and the corresponding ratio to economy-wide growth for each sector. These measures confirm the different magnitude and sectoral distribution of the 2009 and 2020 shocks.

**Table 2:** Short-Term Indicators of Shocks of 2009 and 2020 in OECD-Europe and LAC

		Year-on-year growth rate minimum	Elasticities to economy wide drop (for respective minimums)		
			Manufacturing	Construction	Retail Trade
2009	OECD-Europe	**− 5.1%**	4.1	2.1	0.7
		*Apr-09*	*Apr-09*	*Dec-08*	*Feb-09*
	LAC	− **3.2%**	3.0	1.5	3.1
		*May-09*	*Jan-09*	*Jun-09*	*Apr-09*
2020	OECD-Europe	− **14.2%**	2.1	1.8	1.3
		*Apr-20*	*Apr-20*	*Apr-20*	*Apr-20*
	LAC	− **24.1%**	1.2	2.2	1.6
		*May-20*	*Apr-20*	*Apr-20*	*Apr-20*

		Year-on-.year growth rate for last observation available	Elasticities corresponding to last observation		
2020	OECD-Europe	− **3.9%**	1.0	0.0	− 1.0
		*Aug-20*			
	LAC	− **3.3%**	− 0.2	1.5	0.59
		*Dec-20*			
*Note: Sectoral data from OECD-Europe is available up to December 2020, but the recovery is calibrated using August data to be consistent with the last observation for monthly aggregate GDP evolution*					

		YOY growth rate minimum	Sectoral Ratios corresponding to respective minimums in examined years		
			Manufacturing	Construction	Retail Trade
2020	OECD-Europe	− **8.2%**	1.1	1.2	1.0
		*Apr-20*	*Apr-20*	*Apr-20*	*Apr-20*
	LAC	− **32.0%**	1.1	1.4	1.0
		*May-20*	*May-20*	*May-20*	*May-20*
*Note: LAC is constructed averaging YOY growth rates for Chile, Colombia and Peru*					

		Economy wide	Manufacturing	Construction	Retail Trade
2020	OECD-Europe	− **5.9%**	− **20.8%**	− **15.2%**	− **10.4%**
	LAC	**8.0%**	**6.5%**	− **6.7%**	− **6.4%**

^*^Minimun monthly YOY growth rate for each sector and each variable was taken into account

^*^Some LAC countries report "Trade" GDP data instead of "Retail Trade". Regarding employment data, all countries report Trade, Hotels & Restaurants and Transportation combined

For OECD-Europe, the drop in output in 2020 was almost three times greater than in 2009; for LAC, it was eight times higher. LAC’s lower 2009 drop in output relative to OECD-Europe corresponds with the lower drop in TFP measured in the KLEMS dataset reported in Table 1. Sectoral performance shows that OECD-Europe had higher drops in manufacturing output in both 2009 and 2020, while LAC shows a particularly strong output adjustment in wholesale and retail in both crises and in construction in 2020. Finally, evidence of recovery is still incomplete in both regions, although wholesale and retail in OECD-Europe displays signs of dynamic improvement after mobility restrictions began to be lifted. The image for the first round of the 2020 crisis shows a partial and heterogeneous recovery as mobility was also partially restored in both regions.

**Fig. 3:**
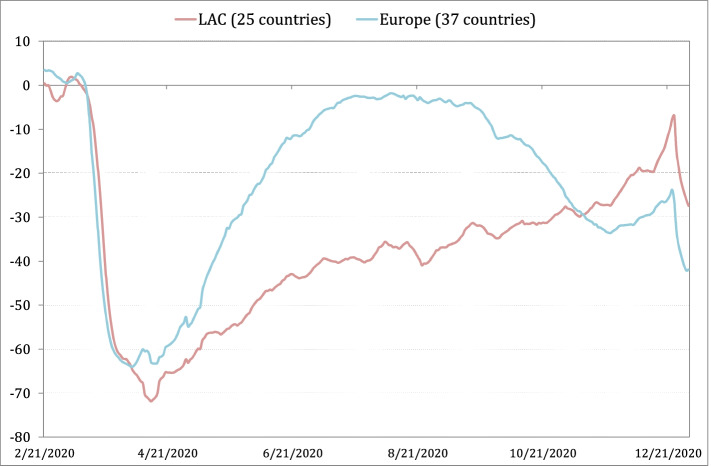
Google mobility: retail & recreation -regional averages % change from baseline, up to 31/12/2020, weekly averages

The bigger drop in output in LAC compared to OECD-Europe in 2020 begs the question of the underlying causes. Methods of decomposition of sectoral shocks such as the VAR approach in Brinca et al. (2020a; b), or the one employed in next section, allocate results between demand or supply shocks, or between sectors in the PVAR impulse-response simulations. But they do not necessarily inform about the ultimate drivers. There are alternative hypotheses in the casual debate that relate the comparatively bad performance of LAC with its sectoral structure (i.e. more biased towards service sectors), its higher degree of informality, and to the deeper fall in mobility due to either stringent lockdowns, or the evolution of the pandemic itself. There are, however, doubts about these channels as the prime causes of the observed size of shocks in LAC relative to OECD-Europe. First, the sectoral structure by itself does not explain where the shocks are located, although it does influence aggregate results. Second, informality does not explain the prevalence or allocation of shocks, as a similar supply-side shock based on constraints to mobility, for example, would impact a sector the same regardless of the formal/informal nature of labor contracts, except that informal environments can bypass lockdowns more easily (which, in turn, would go against explaining a higher relative drop in output in LAC). Third, there is an empirical issue about whether LAC suffered more severe restrictions to mobility, with evidence showing a deeper fall in aggregate mobility in LAC during 2020. However, differences are significantly less important in mobility affecting retail and recreation (R&R) activities in LAC compared to OECD-Europe. Figure 3 shows that the initial drop in R&R mobility in LAC and OECD-Europe was similar in magnitude and the divergence later, relates in part to the different seasons in the northern and southern hemispheres, and the asynchronous COVID-19 phases. There is a different explanation of what is behind the observed shock differential between LAC and OECD-Europe that is in line with the focus on productivity shocks, and the role of infrastructure in this paper. In the case of trade and hospitality, a better infrastructure insofar as communications (digitalization, transport, and storage) may have made a difference between LAC and OECD-Europe given their importance in the management of transactions that economize on interpersonal contacts. This is an avenue for further research to be pursued with alternative datasets. In the next section, we implement a PVAR approach to explore the interaction of sectoral short-run shocks, allowing us to make some reasonable conjectures about the likely effects of sectoral productivity shocks.

## A PVAR of Sectoral Productivity Growth Rates

Given the nature of the shocks experienced since the start of the COVID-19 pandemic, it is not surprising that we should observe different impacts on sectoral productivity. We want to learn how, and to what extent, the shocks in the earlier episodes and in the more sensitive sectors in the current crisis exert effects on other sectors. Due to the particular nature of these shocks, the approach should be different from earlier analyses of the long-run elasticities of productivity shocks on other sectors (e.g., Ahumada and Navajas 2019). Because of that, and to avoid short-run endogeneity, our analysis is based on a PVAR estimation of productivity growth rates in 24 countries over 1995–2015, using data from the KLEMS database. We start by estimating the global panel and then compare this joint estimation with the LAC case. This approach allows us to learn the interaction of sectoral short-run shocks, allowing us to make some reasonable conjectures about the likely effects of sectoral productivity effects ascribable to the COVID-19 crisis, focusing all the while on regional differences.

### The Estimation Approach

For the purpose of evaluating sectoral (*s*) effects originating in productivity changes in the (*j*) sectors most affected by the pandemic, conditional (single-equation) models cannot be properly estimated, even when instrumental variables are used for the normalization (*s* over *j*). This is due to high sectoral correlation when annual data are used. To avoid this problem, we adopt a PVAR approach to examine these effects based on the innovations obtained from the VAR estimation, since we are interested in gauging the interactions of sectoral shocks rather than developing a structural model. Given the database, we estimate a PVAR, which is represented as follows^6^:

1 $$y_{it} = y_{it - 1 } A + \mu_{i} + e_{it}$$

*i* = *1,…,n* (countries in our study) and *t* = *1,..,T* (years); $y$ is a *1 *×* k* vector of endogenous variables (here sectoral productivities) and ${\upmu }_{i}$ and ${e}_{it}$ are *1xk* vectors of (country) individual effects and idiosyncratic errors, respectively (for simplicity, one lag is assumed without loss of generality). The *kxk* matrix A is composed of the reduced-form parameters to be estimated. We assume these parameters to be the same across countries and, as shown in Eq. (1), cross-sectional heterogeneity (and dependence) is modeled only as panel-specific fixed effects (${\upmu }_{i}$). Because these effects and lagged dependent variables are included in the right-hand side of the system of equations, given the T size, GMM estimates are used to avoid Nickell biases that might arise after variables are transformed to remove ${\upmu }_{i}$.

In order to analyze the response of one variable (*s*) in the system when another (*j*) is subject to a shock, the moving average (MA) representation is generally used. After removing the fixed individual effects (${\upmu }_{i}$) from Eq. (1) the MA representation with parameters φ is

2 $$y_{it} = \varphi \left( L \right) e_{it }$$

where

$$\varphi \left( L \right) = I_{k} + \varphi_{1 } L + \varphi_{2 } L^{2} + \cdots$$

In this approach it is assumed that the innovations ${e}_{it}$ are serially uncorrelated (with their own lags) but, since they are contemporaneously correlated (among variables) ${E [e}_{it}^{^{\prime}}{e}_{it}]= \Omega$, these innovations are transformed, usually, by the Cholesky decomposition, which imposes a recursive ordering structure (${P. P}^{{^{\prime}}}=\Omega$), to obtain orthogonalized innovations,${u}_{it}$,

3 $$u_{it} = { }e_{it} P^{ - 1}$$

where each element ${u}_{jit}$ of the vector ${u}_{it}$ is interpreted as the residual from the projection of ${e}_{jit}$ on ${u}_{1it}$,${u}_{2it}$, …,${u}_{j-1it}$.^7^

Furthermore, it is also common to calculate $${v}_{it}$$ which is just $${u}_{it}$$ divided by its standard deviation ($${d= {Var({u}_{it}) }^{1/2}}$$).^8^ Then, using an impulse-response function, we observe the marginal effect of an innovation impulse $$({v}_{jit )}$$ on the endogenous ($${y}_{sit+h )}$$ for *h-*periods ahead, holding all other innovations at all dates constant.^9^

$$\frac{{\partial y_{sit + h } }}{{\partial v_{jit } }} = \frac{{\partial y_{sit + h } }}{{\partial u_{jit } }}d_{jj}$$

Moving to an orthogonalized impulse-response function (OIRF), we calculate the consequences of ${y}_{sit+h}$ on the forecast using the “new” information, which is different for each variable *j* (see Hamilton 1994: 322). Using ${v}_{jit}$, we expressed the innovation as if ${y}_{jit}$ were to increase in one standard deviation. We note that from this equality it is possible to obtain a one-unit increase in the *j*th variable’s (orthogonalized) innovation, ${u}_{jit}$ dividing the left-hand side by ${d}_{jj}$.

Finally, we compare the recursive ordering in this approach with a structural representation of the PVAR,

4 $$y_{it} B_{0} = y_{it - 1 } B_{1} + \mu_{i}^{*} + u_{it}$$

where ${B}_{0}$ contains the contemporaneous effects. The PVAR in reduced form then becomes Eq. (1) when

5 $$\begin{array}{rcl} A & =& B_{1} B_{0}^{ - 1} \\ \mu_{i} & =& \mu_{i}^{*} B_{0}^{ - 1} \\ e_{it} & =& u_{it} B_{0}^{ - 1} \quad {\text{and}} \\ u_{it} & =& e_{it} B_{0} \\ \end{array}$$

Comparing (3) with (5), we assume a recursive ordering for the contemporaneous effects when ${B}_{0}{= P}^{-1}$, the same identification we used when calculating the OIRFs.

A feature of this study allows us to apply the recursive ordering. We know that the shocks during the COVID-19 pandemic are likely to start in the hospitality and wholesale and retail trade sectors, and that construction and manufacturing sectors are also among the most-affected sectors (see Brinca et al. 2020a and Sect. 2 above). Thus, we can order the variables starting from these supposedly more exogenous sectors—wholesale and retail, hotels and restaurants (*thr*); construction (*con*); manufacturing (*man*)—to the rest of sectors. For the latter we follow the ranking of least to most capital intensive as estimated in Cavallo and Powell (2021).

For the estimation of the PVAR, the variables are sectoral labor productivities expressed as log differences. No exogenous variables were considered. A sensitivity analysis was also performed using total factor productivities (Online Appendix D), which show minor differences in coefficient values and significance owing to the use of labor productivity figures (Table 3). The PVAR was estimated using two lags.^10^

**Table 3:** Orthogonalized impulse-response functions for sectoral growth rates in labor productivity

Response variable & forecast horizon (years)	Impulse variable					
	dly_thr		dly_man		dly_con	
	*OECD & LAC*	*LAC*	*OECD & LAC*	*LAC*	*OECD & LAC*	*LAC*
**dly_thr**						
0	0.0472*	0.0704*	0.0174*	0.0234*	0.0109*	0.0174*
1	− 0.0043	− 0.0111	0.0041	− 0.0003	− 0.002	− 0.0092
2	0.0009	0.0037	− 0.0071	− 0.011	0.0042	0.0082
3	0.0009	0.005	0.0036	0.0099	0.0018	0.004
4	0.0000	− 0.0019	− 0.0017	− 0.0044	− 0.0014	− 0.0043
5	− 0.0004	− 0.0008	0.0007	0.0014	0.0007	0.0024
**dly_con**						
0	0.0145*	0.0225*	0.0261*	0.0507*	0.0630*	0.0910*
1	0.0036	0.0073	− 0.0062	− 0.0092	0.0009	− 0.0044
2	− 0.0017	− 0.0037	− 0.0003	0.0019	0.0019	0.0014
3	0.0008	0.0012	− 0.0021	− 0.0038	− 0.0008	− 0.0024
4	− 0.0009	− 0.0015	0.001	0.0027	0.0009	0.0015
5	0.0004	0.0006	− 0.0005	− 0.0012	− 0.0002	− 0.0008
**dly_man**						
0	0.0219*	0.0243*	0.0596*	0.0731*	0.0247*	0.0407*
1	− 0.0069	− 0.0163	− 0.0094	− 0.0257	− 0.0083	− 0.0167
2	0.0006	0.0022	0.0032	0.006	0.0036	0.0098
3	0.0013	0.0034	− 0.0016	− 0.0033	− 0.003	− 0.0062
4	− 0.0015	− 0.0041	0.0005	0.0011	0.001	0.0017
5	0.0007	0.0023	− 0.0006	− 0.0006	0.0001	0.0005
**dly_tsc**						
0	0.0208*	0.0312*	0.0213*	0.0279*	0.0169*	0.0295*
1	− 0.0076	− 0.0156	− 0.0046	− 0.0182	− 0.0084	− 0.0195
2	0.0022	0.006	− 0.0031	− 0.0036	0.0089	0.018
3	0.0012	0.007	0.0021	0.0089	0.0006	0.0006
4	0.0001	− 0.003	− 0.0018	− 0.0045	− 0.0009	− 0.0044
5	− 0.0002	− 0.0003	0.0004	0.0009	0.0005	0.0023
dly_utl						
0	0.0353*	0.0593*	0.0092*	0.0125	0.0054	0.0161
1	− 0.0011	− 0.0037	0.0269*	0.0399	0.0158	0.0211
2	0.0108	0.0126	− 0.0052	− 0.024	− 0.0033	− 0.0055
3	− 0.0032	− 0.0014	0.0057	0.0105	0.0039	0.0106
4	0.0022	0.0058	− 0.0041	− 0.0071	0.0007	− 0.0001
5	− 0.0004	− 0.0008	0.0023	0.0059	0.0013	0.0031
dly_fire						
0	0.0271*	0.0511*	0.0016*	0.0053*	0.0044	0.0086
1	− 0.007	− 0.0115	0.0036	0.0064	0.0011	− 0.0035
2	0.0061	0.012	− 0.0101	− 0.0149	0.0015	0.0021
3	− 0.0007	0.0006	0.0045	0.011	0.0011	0.0022
4	0.0000	− 0.0013	− 0.0033	− 0.0068	− 0.001	− 0.0036
5	− 0.0004	− 0.0005	0.0017	0.004	0.001	0.0032

^*^significant at 5%

Given our interest in the transmission of the shocks, we compute OIRFs with Monte Carlo–simulated SE^11^ to calculate confidence intervals and thus to detect significant effects.

### Results

Table 3 and the figures in Online Appendix C present the results. In all cases the OIRFs indicate (i) only a significant immediate positive (of the same sign) of the variable experiencing the initial shock, (ii) that the impact effects on the growth rates are not reversed in following years, and (iii) that the shocks in LAC are greater than those appearing in the joint estimations.

In the case of an exogenous one-standard deviation shock (0.047 percentage points [pp]) in the domestic trade and hospitality sector (*thr*), the impacts on the growth rates of other sectors are between 0.015 pp for construction (*con*) to 0.035 pp for utilities (*utl*). These are effects for the whole sample and all countries. For LAC, with a shock in the growth rate of labor productivity in *thr* equal to one standard deviation (of 0.07 pp), the impacts are 0.023 pp and 0.059 pp on the same sectors. These two one-standard-deviation shocks for the whole sample and for LAC were similar in magnitude to the actual cross-country average decrease in *thr* productivity in the previous 2009 global crisis, both globally and in LAC (0.051 and 0.083 pp, respectively). However, the relevant result is the significance of the estimated impulse-response effects. As with the magnitudes, we suspect that this is a conservative approximation for the COVID-19 crisis; the effects may be larger, given the greater magnitude of the *thr* shock in 2020 in both OECD and LAC.

In the case of an exogenous shock of one standard deviation (0.06 pp) in the manufacturing sector (*man*), the impacts on the sectoral growth rates are lower than for *thr*. They are between 0.002 for finance, insurance, and real estate (*fire*) and 0.026 for *con*. In the case of LAC, the impacts of a shock of one standard deviation in the *man* growth rate (of 0.073 pp) range from 0.05 pp (*fire*) to 0.05 pp (*thr*), and they are not significant in the case of *utl*. Finally in the case of an exogenous shock of one standard deviation (0.063 pp) in the construction sector, the impacts on the sectoral growth rates are also lower than in the case of *thr*, standing between 0.01 (*thr*) and 0.025 (*man*) and not significant in the case of *fire* and *utl*. In LAC, the impacts are, for a shock of one standard deviation (0.09), between 0.02 (*thr*) and 0.04 (*man*) and not significant in the case of *fire* and *utl.*

Our econometric estimates show that productivity shocks in sectors recognized as having important roles in the 2009 and 2020 crises have both direct and indirect short-run effects. These shocks are particularly relevant to the task of simulating the effects of the COVID-19 crisis, most notably in the case of *thr*, because the estimated effects are significant, and because the magnitude of the COVID-19 shocks in the sector is at least twice as large as the one we used to estimate impulse-response sectoral effects. The exogenous shocks to labor productivity in *thr* have a somewhat larger and more diffuse effect on other sectors than do those in *man* and *con*. Except for *fire* and *utl,* the other sectors show interaction effects. Note that *con* and *thr* have a larger labor component in developing countries, which should also be considered when analyzing the transmission of shocks. Finally, the short-run nature of the previous analysis should not be ignored. To understand the postcrisis effects in the medium and long term, where infrastructure may play a key role, the models should include both short- and long run effects.

## Magnitude of Combined Sectoral Shocks

The foregoing impulse-response exercise assumes that shocks in trade, hotels and restaurants (*thr* in the definition used in Table 3); construction; and manufacturing make up a reasonable representation of the bulk of COVID-19 shocks on productivity. Table 4 summarizes the significant coefficients of the shocks in these three sectors, along with the proposed or assumed orders of magnitude. Values of estimated coefficients for LAC are distinguished from those of the sample as a whole. The assumed impulses in all three sectors have a direct impact on aggregate productivity and second-round effects, through the response in other sectors. For instance, and due to the chosen ordering of sectoral shocks, the productivity shocks of trade and hospitality services have, apart from their direct effect, indirect effects on all other sectors evaluated in the PVAR. By contrast, shocks in construction affect the manufacturing and transport sectors, while those in manufacturing affect transport for the whole sample and utilities in LAC. All coefficients are expressed in absolute growth rates. Table 5 represents, purely for illustrative purposes, the magnitude of the elasticities of each sectoral productivity effect after a change in each of the sectors where shocks occur, as explained in Sect. 3. For example, the assumed impulse shock of 7 percent in trade and hospitality services in LAC elicits a response in several sectors, with elasticities of between 0.32 for construction and 0.84 for utilities.

**Table 4:** Shock Coefficients of Impulse-Response Function

Response sector	Impulse sector					
	Trade, hotels, and restaurants		Construction		Manufacturing	
	OECD and LAC	LAC	OECD and LAC	LAC	OECD and LAC	LAC
Construction	0.0145	0.0225	0.0613	0.0882		
Finance, insurance, and real estate	0.0271	0.0511				
Manufacturing	0.0219	0.0243	0.0202	0.0358	0.0516	0.0589
Trade, hotels, and restaurants	0.0472	0.0704				
Transport, storage, and communications	0.0208	0.0312	0.0125	0.0224	0.0108	
Utilties	0.0353	0.0593				0.0372

Only significant values are shown, all corresponding to contemporary effects, with the exception of the Manufacturing shock over Utilities, which corresponds to the first lag effect

**Table 5:** Elasticities of Impulse-Response Function

Response Sector	Impulse Sector					
	Trade, hotels, and restaurants		Construction		Manufacturing	
	OECD and LAC	LAC	OECD and LAC	LAC	OECD and LAC	LAC
Construction	0.31	0.32	1	1		
Finance, insurance, and real estate	0.57	0.73				
Manufacturing	0.46	0.34	0.33	0.41	1	1
Trade, hotels, and restaurants	1	1				
Transport, storage, and communications	0.44	0.44	0.20	0.25	0.21	
Utilties	0.75	0.84				0.63

Only significant values are shown, all corresponding to contemporary effects, with the exception of the Manufacturing shock over Utilities, which corresponds to the first lag effect

Adding up all sectoral productivity shocks weighted by the labor share of each sector yields the effects for the economy, which can be broken down into direct and indirect effects. Table 6 illustrates these results. “Total effects” refers to the sum of direct and indirect effects, while the economy-wide aggregate effect is the sum of all three total effects. Combined productivity shocks of one standard deviation in trade and hospitality, construction, and manufacturing in LAC add up to a shock of 4.9 percent on aggregate productivity (= 2.8 + 0.8 + 1.3); the effect is 3.5 percent for the entire sample. About two-thirds of the aggregate effect is due to direct effects in LAC (60 percent for the whole sample). The distribution of the effect across sectors is shown in the lower panel of Table 6.

**Table 6:** Direct and Indirect Effects of Combined Sectoral Shocks

	Country group	Initial shock on THR sector			
		Trade hotels, and restaurants	Manufacturing	Construction	
Total effect	*OECD and LAC*	**2.0%**	**0.8%**	**0.8%**	
	*LAC*	**2.8%**	**0.8%**	**1.3%**	
Direct effect	*OECD and LAC*	1.0%	0.7%	0.4%	
	*LAC*	1.8%	0.8%	0.7%	
Indirect effect	*OECD and LAC*	0.9%	0.1%	0.4%	
	*LAC*	1.1%	0.0%	0.6%	
Indirect effect disaggregation: % contribution of each sector					
Trade, hotels, and Restaurants	*OECD and LAC*				
	*LAC*				
Construction	*OECD and LAC*	11.2%			
	*LAC*	15.8%			
Manufacturing	*OECD and LAC*	30.5%		73.9%	
	*LAC*	28.8%		76.0%	
Transport, storage, and communications	*OECD and LAC*	16.6%	100%	26.1%	
	*LAC*	18.7%		24.0%	
Utilities	*OECD and LAC*	3.5%			
	*LAC*	3.9%	100%		
Finance, insurance, and real estate	*OECD and LAC*	38.3%			
	*LAC*	32.8%			

**Fig. 4:**
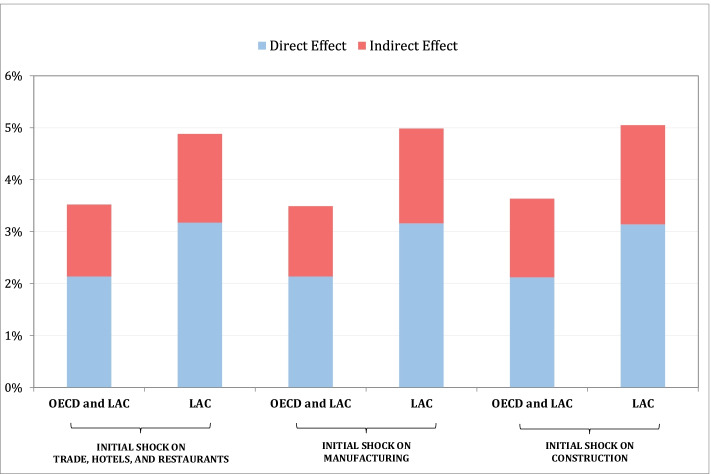
Decomposition of aggregate interactive shock by country group and magnitude of initial shock

**Fig. 5:**
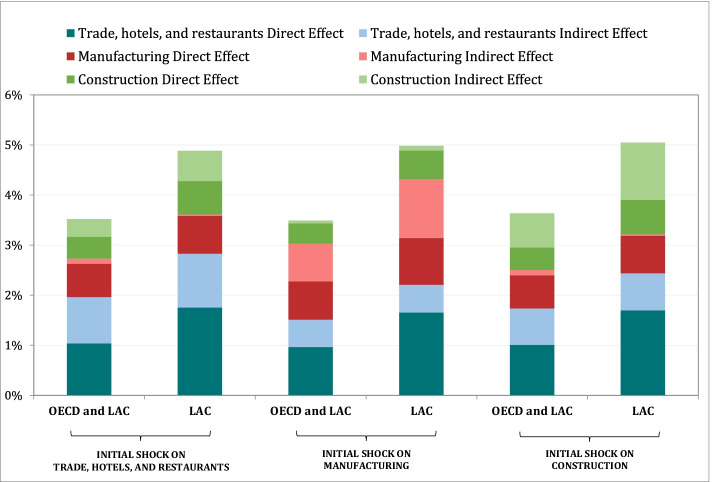
Decomposition of shocks by sector, country group, and magnitude of initial shock

The sensitivity of the results shown in Table 6 to a different ordering of the sectors in the impulse-response simulation is illustrated in Fig. 4. As previously noted, the ordering of the sectors matters for an impulse-response simulation. Our chosen ordering—starting with trade and hospitality services—is based on the observed nature and magnitude of COVID-19 shocks and their relationship to the capital intensity of sectors. But with different orderings, the aggregate results are more or less preserved (Fig. 5). The distribution of effects across sectors does change and depends more on the effects in the sector where the shock is initiated. These results indicate that the aggregate economy-wide magnitude of the simulated productivity shocks does not change much when changes are made in the assumed ordering of sectoral shocks.

## Compensatory Effects from Infrastructure Productivity Improvements

Having estimated the impacts of productivity shocks in the sectors most affected by the COVID-19, we turn now to so-called compensatory effects that could reverse those impacts. The natural candidates are improvements in infrastructure productivity and a deepening of capital in the sectors that suffered the shocks most intensively. Once again, wholesale, retail, and hospitality appears to be the sector best suited to a study of the compensatory effects of improvements in infrastructure productivity. This is so for two reasons: first, the empirical evidence shows that this sector absorbed much of the shock from the pandemic and remains the most affected by its successive waves. Manufacturing and construction, by contrast, have recovered strongly as economies reopened. Second, while we develop a general approach to estimate the effects of infrastructure on sectoral productivity across sectors, we find that the results are less robust for manufacturing and construction than for wholesale, retail, and hospitality*.*^12^ We perform the analysis using the panel of countries studied in Sect. 2, focusing on the long-run determinants and identifying relevant infrastructure-related sectors that may contribute to productivity growth.

### Econometric Approach

Our approach is similar to the one proposed in Ahumada and Navajas (2019). We begin with unrestricted models of labor productivity (output per worker in logs, $y$) in sector *s* (for example wholesale, retail, restaurants and hotels) taking as explanatory variables the capital–labor ratio of the sector (${k}_{s}$), the labor productivity of the three infrastructure sectors “r” (${y}_{utl}, {y}_{con},{ y}_{tsc}$), and the capital per worker in those sectors (${k}_{utl}, {k}_{con},{k}_{tsc}$) so as to distinguish productivity effects from capital-stock effects. The underlying assumption is that infrastructure TFP enters the production function of sector *s*. This effect (or others stemming from labor or capital productivity in the infrastructure sector) could exert a compensatory effect on productivity in sector *s*.

We also include in the unrestricted model two control variables ($x$)—one a measure of trade openness (the country share in the sample’s total exports plus imports); the other a proxy for human capital. To evaluate country heterogeneity, we include fixed effects through 24 dummy variables (one for each country), time effects (years), and outliers (impulse dummies for a specific country–year observation). To handle this large information set, we relied on an automatic algorithm (*Autometrics,* see Doornik, 2009, and Hendry and Doornik, 2014) to select the relevant variables. The algorithm uses a tree search to discard paths from the initial unrestricted model, based on ordered squared t-statistics, for a given a p-value.^13^ We note that, by including country dummies without restriction (instead of using deviations from country means as in the usual fixed-effect estimation), we can evaluate the intercept country heterogeneity by observing the dummies selected by the algorithm.

We consider the possibility of unit roots and evaluate cointegration by formulating the unrestricted model, expressing the dependent variable in terms of log differences and the explanatory variables in both log levels and log differences, as suggested by Bardsen for time series (reported in Banerjee et al. 1993) and Westerlund (2007) for panel data. Therefore, the initial unrestricted model takes the following form:

6 $$\begin{array}{rcl} \Delta y_{s,it} & =& \alpha_{i} + \gamma_{t} + \delta_{s} y_{s,it - 1} + \beta_{s,utl} y_{utl, it - 1} + \beta_{s,con} y_{con, it - 1} \\ &&  + \beta_{s,tsc} y_{tsc, it - 1} + \varphi_{s,utl} \Delta y_{utl, it} + \varphi_{s,con} \Delta y_{con, it} \\ && + \varphi_{s,tsc} \Delta y_{tsc, it} + \theta_{s,utl} k_{utl, it - 1} + \theta_{s,con} k_{con, it - 1} \\ && + \theta_{s,tsc} k_{tsc, it - 1} + \theta_{s,s} k_{s, it - 1} + \lambda_{s,utl} \Delta k_{utl, it} \\ && + \lambda_{s,con} \Delta k_{con, it} + \lambda_{s,tsc} \Delta k_{tsc, it} + \lambda_{s,s} \Delta k_{s, it} + x^{\prime}_{it - 1} \phi_{s} \\ && + \Delta x^{\prime}_{it} \tau_{s} + \varepsilon_{s,it} \quad i = 1, ..,N;\quad t = 1, \ldots ,T \end{array}$$

where *i* indicates each country and *t* each year of the panel for sector *s*. In the first row we have the coefficient of the country, time effects, and the long-run effects of labor productivities given by an adjustment coefficient ${\delta }_{s}$ (which is expected to be significantly negative under cointegration) along with the long-run infrastructure sector elasticities given by the negative value of ${~}^{\beta _{s,utl}}\!\left/ \!\!{~}{{\delta }_{s}}\right. {~}^{,{ \beta }_{s,con}}\!\left/\!\!{~}{{\delta }_{s}}\right. {~}^{, {\beta }_{s,tsc}}\!\left/ \!\!{~}{{\delta }_{s}}\right.$.^14^ The next row indicates the impact effects of changes in infrastructure productivities. The third and four rows include parameters for the long- and short-run effects of capital per worker in infrastructure and sector *s*. The last row accounts for the control variables in vector $x^\prime$. All variables are in logs.

From the log function in Eq. (6) we can also obtain the effects of capital productivity in the infrastructure sector. In this case, the estimates should not reject the hypothesis that ${\beta }_{s,r=}{- \theta }_{s,r}$ for *r* = *utl, tsc, con,* because when that hypothesis holds the corresponding effects become ${{\beta }_{s,r} y}_{r, it-1}- {{\theta }_{s,r} k}_{r, it-1}= { \beta }_{s,r} (ln$ (Y/L)—$ln$ (K/L) = ${\beta }_{s,r} (ln$ (Y/K). Therefore, the estimate of ${\beta }_{s,r}$ is the elasticity of capital productivity of infrastructure sector *r*.

By nesting levels and differences, Eq. (6) allows us to have variables that enter the model only in the long run, only in the short run, or in both. The advantage of estimating this type of model is that it can be easily reparametrized as an error-correction model, which includes growth rates and deviations from the long-run relationship. For example, when we observe only a long-run effect of infrastructure sector *r* on productivity in sector *s*, the restricted Eq. (6) would have the following error-correction representation:

7 $$\Delta y_{s,it} = \alpha_{si} - \delta_{s} \left[ {y_{s,it - 1} - \beta_{s,r}^{*} y_{r, it - 1} } \right] + \varphi_{s,r} \Delta y_{r, it} + \varepsilon_{s,it}$$

where $${\beta }_{s,r}^{*}={~}^{{\beta }_{s,r}}\!\left/ \!\!{~}{{\delta }_{s}}\right.$$

If the variables are first-order integrated, we can test whether this long-run relationship is a cointegration vector by evaluating the significance of the t-statistic of the lagged explained variable (of the estimated coefficient of ${\delta }_{s}$). Although the distribution of this statistic is nonstandard when there is no cointegration, the critical values derived from the response function in the Monte Carlo study of Ericsson and MacKinnon (2002) can be used to test cointegration.^15^

We start by assuming that infrastructure sector labor productivities are exogenous and then test the assumption in two ways. After infrastructure variables are entered contemporaneously into the selected model (as log differences), we re-estimated the model using instrumental variables. Our main assumption is that the capital-per-worker variables of the infrastructure sectors are exogenous and can therefore be used as valid instruments. However, in the case of variables with unit roots (see Hendry, 2007). and when we focus on the long-run representation, we must be sure that the error-correction term does not enter the marginal model. This requires that no level of sector *s* enters into the equation of an infrastructure sector. We tested this and could not rule out long-run weak exogeneity (see Juselius 2006) in the models studied.

One difference with Ahumada and Navajas (2019) is the time dimension of our panel. A sample size of T = 20 after lagging variables is a borderline case between small and large samples for dynamic models with fixed effects. For example, Beck and Katz (2011) consider that the Nickell bias becomes small for 20 or more time observations, based on a previous Monte Carlo study they conducted. Their results show that ordinary least squares estimates of a simple least square dummy variable (LSDV) model are similar to those obtained using Kiviet and Anderson-Hsiao estimators. This allows us to derive consistent unrestricted parameter estimators in Eq. (6) so as to start the selection algorithm. However, as the bias may also depend on the size of autoregressive coefficients and the estimated variance of the dynamic model, we compare the estimated long-run elasticities derived by the algorithm from the selected model with those obtained by correcting the LSDV estimates for the (order 1/T) bias, as suggested by Kiviet and implemented by Bruno (2005), and with the bootstrapped estimates of dynamic panels (De Vos et al. 2015).^16^

### Results

The model selected by Autometrics for the panel of 24 countries over the period 1996–2015 is shown in Table 7.^17^ Definition of the variables is similar to that used previously for sectors, while *l* stands for logs and *d* for first differences. Online Appendix Table A2 provides more details on variables and sources.

**Table 7:**
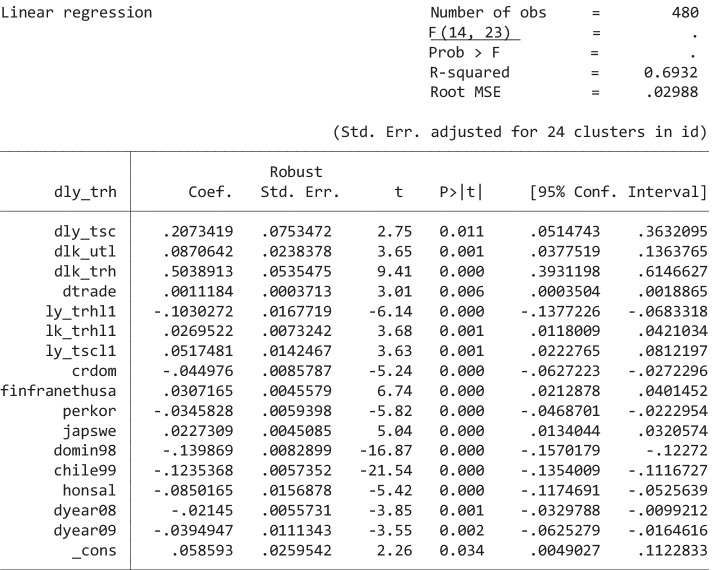
Model Used for Unrestricted Estimations of Eq. (6)

The model in Table 7 yields significant short-run effects from the log differences of transport labor productivity, capital per worker of utilities and the focus sector, and the change in trade openness.^18^ Regarding country fixed effects, we can separate groups of low and high productivity that reduce the 24 parameters (one for each country) to 6. We also observe that only 2008 and 2009 were significant as time effects and outliers associated with the earthquakes in Dominican Republic in 1998 and Chile in 1999.

As far as long-run effects are concerned, the capital–labor ratio in the focus sector and labor productivity in the transport sector are parts of the cointegration relationship (as tested by the Ericsson–Mackinnon rule), which is^19^

8 $$\begin{array}{rcl} Ly\_thr & = & 0.26 \, lk\_thr \, + \, 0.50 \, ly\_tsc \\ && \left( {0.058} \right) \left( {0.102} \right) \end{array}$$

This conditional model was validated by testing that the *thr* labor productivity was not significant when the dependent variable was the log difference of transport labor productivity in the model in Table 7.

The long-run estimates in Eq. (8) show that the long-run elasticity of labor productivity in trade and hospitality is 0.5 with respect to labor productivity in transport; and near 0.3 with respect to the capital per worker ratio in trade and hospitality. Since these long-run parameters are obtained from the short-run estimates, the standard errors reported in Eq. (8) are calculated from an approximation to their long-run variance.^20^

Table 8 shows the same long-run elasticities calculated using LSDV bias-corrected and bootstrapped estimates along with the confidence interval of the long-run estimates from Eq. (8), where LowL and UppL indicate lower and upper limits of the 95 percent interval.

**Table 8:** Comparison of Bias-Corrected Estimates and Ordinary Least Squares Confidence Intervals of Long-Run Elasticities

LR elasticity	LSDVcorrected	bootstrapped	LowL	UppL
lk_trh	0.36	0.36	0.15	0.38
ly_tsc	0.36	0.31	0.30	0.71

Because the two corrections of the long-run elasticity estimates are within the 95 percent confidence interval of the estimates from Table 7 (being closer to the upper limit in the case of the focus sector’s capital elasticity and to the lower limit in the case of transport productivity), we can proceed to compute the compensatory effects required in transport labor productivity using both the point estimates in Eq. (8) and the limits of the reported confidence intervals of the long-run elasticity.

### Magnitude of Compensatory Effects from Infrastructure Productivity Improvements

The elasticity interval of long-run labor productivity in transport on the productivity of wholesale, retail, and hospitality services enables us to estimate the magnitude of improvement in the former needed to make up for the negative productivity shock engendered in the latter by the COVID-19 pandemic. This is simply approximated by the ratio of *z/(1* − *z)* and the estimated elasticity range in Table 8, where *z* is the size of the shock in the wholesale, retail, and hospitality sector observed after the COVID-19 crisis. Thus, a long-term productivity improvement in transport infrastructure will help to restore the productivity lost in the wholesale, retail, and hospitality sector.^21^ Given the range of the estimated interval for the elasticity estimated in Table 8 (0.3 to 0.71), the improvement needed in the productivity of transport ranges from 7 percent to 16.5% for the entire sample to 10% to 25% for the LAC economies alone. The required improvements are large, particularly in the case of the LAC countries, despite their extending over the long run. At the historical rate of labor productivity improvement in the transport sector observed in our sample (2.3% per year for the whole sample and 2.9% for LAC), the required gain would take several years. Therefore, the rate of improvement in the productivity of transport infrastructure must be accelerated. This turns our attention to ways to spur infrastructure productivity by means of fiscal, institutional, and other supporting policies.

## Conclusions

This paper studied shocks to sectoral productivity induced by the COVID-19 pandemic, the aggregate impact of those shocks, and the possible compensatory effects of improving productivity in infrastructure-related sectors. We used a KLEMS annual dataset from 1995 to 2015 for a group of OECD and LAC countries. The dataset was complemented with high-frequency data of sectoral output and labor during 2020 to define a likely configuration of sectoral shocks after the COVID-19 pandemic. The analysis, complemented by other available evidence, led us to select three one-digit sectors—wholesale, retail, and hospitality; manufacturing; and construction—as the main candidates on which to model productivity shocks.

After estimating a PVAR of sector-specific rates of growth in labor productivity to characterize the nature and size of shocks in the 24 OECD and LAC countries in our sample, we ran an impulse-response simulation of shocks in the chosen sectors.

We separated estimates for the whole sample of OECD and LAC countries, and for LAC countries alone, as the shocks in the latter were larger. We also computed aggregate, direct, and indirect effects of labor productivity losses. On aggregate, shocks in these three sectors amounted to a 4.9% shock on overall labor productivity in the LAC economies, and 3.5% for the whole sample.

Finally, we assessed the degree of improvement in the productivity of infrastructure-related sectors that might be required to compensate for the losses caused by the shocks caused by COVID-19. Following an approach that encompasses labor and capital productivity shocks in infrastructure, along with capital deepening, we applied an econometric modeling framework to assess the long-run relationship between productivity in the wholesale, retail, and hospitality sector, the sector most affected sector by COVID-19, and that in infrastructure-related sectors. We found that the increases in the productivity of transport sector infrastructure (which given the level of aggregation in our data includes telecommunications and storage) that would be required to compensate for the COVID-19 productivity shocks would be much larger than the historical rates of improvement observed in our sample, particularly the LAC countries.

This conclusion draws attention to the need for selective policy actions that operate through improvements in the regulatory compact of infrastructure services, a point stressed in recent reviews of the scope for improving infrastructure service performance in the LAC region, both generally and in relation to COVID-19 (Cavallo et al. 2020; Izquierdo et al. 2020; Powell and Cavallo 2020). Complementarily, changes in fiscal and labor policy and regulation to facilitate the reallocation of employment in the service sectors should be considered.

The analysis in this paper is consistent with a scenario where COVID-19 shocks are not permanent, and therefore, there will be recovery. But even if the recovery in rates were complete, there will still be a loss in levels that requires compensation. Fully recovering growth rates do not invalidate the compensatory exercise performed in this paper. The estimates for compensatory effects were obtained from a drop in the level of productivity, rather than from a permanent fall in growth rates. While we may have overestimated the effort coming from infrastructure if alternative (more optimistic) productivity scenarios were to materialize, the unique role of infrastructure, in particular transport, communication, and digitalization, is part of the consensus between pessimist and optimist visions of the aftermath of the pandemic.

Further work along the lines of this paper is constrained by the limitations of the datasets needed to better assess the impulse response of shocks and the possible compensatory effects of productivity improvements in infrastructure services. One promising line would be to look at those countries in the KLEMS sample where the necessary sectoral disaggregation is available. The disaggregation is possible for the OECD countries but not, given the current state of the LAKLEMS dataset, for LAC (except for Mexico). Another avenue would be to study individual country models. One likely case for the LAC region is Mexico, where recent results on the sectoral productivity effects of improvements in infrastructure productivity (Ahumada et al. 2021) would seem to permit an extension of the modeling lines of this paper. A third line would exploit micro-datasets at the level of firms or establishments that may make it possible to observe intrasectoral shocks and the effects of improvements in infrastructure services.

## Supplementary Information

Below is the link to the electronic supplementary material.

Supplementary file1 (DOCX 255 kb)

## Footnotes

1 The association between low capital intensity and low total factor productivity was examined by Cavallo et al. (2013) using a model where sectoral investment across countries is negatively affected by relative price volatility, a common feature in LAC economies.

2 In the case of LAC, Teles and Mussolini (2012)—using measures of physical infrastructure in Argentina, Brazil, Chile, and Mexico between 1950 and 2000—do not find a long-run relationship between TFP at the economy-wide level.

3 More recently, Ahumada et al. (2021) extend this approach for Mexico, using the KLEMS-INEGI dataset. KLEMS-INEGI is disaggregated enough to permit a high degree of specificity about the infrastructure subsectors (for example, road freight within the transport sector or civil engineering within the construction sector) whose productivity affects which subsectors of agriculture, manufacturing, or services (for example, crops, automobiles, and hotel services).

4 As in Ahumada and Navajas (2019) we include the three LAC countries in the OECD (Chile, Colombia and Mexico) with the LAC group. Other LAC countries with sufficient information available in the in the LAKLEMS dataset are Costa Rica, El Salvador, Honduras, Peru, and the Dominican Republic. The non-EU countries are Australia, Canada, Japan, South Korea, and the United States. For details, see Online Appendix A.

5 The category of wholesale and retail trade, hotels, and restaurants, is often referred indistinctively in this paper as “trade and hospitality,” “trade, hotels, and restaurants,” or “domestic trade.” The sector accounts for almost a quarter of employment, on average, in the LAC countries reported in the KLEMS dataset, while construction commands a share of less than 8% in total formal employment. The OECD figures for trade, hotels, and restaurants are much lower; for construction they are about the same. When the figures are adjusted for some proxy of informality (which, in the KLEMS dataset, can be approximated by the percentage of the sectoral workforce who are not formal employees) the differences in the trade, hotels, and restaurants sector are larger.

6 The following representation and discussion are based on Hamilton (1994, Chapters 10 and 11) for time series and Abrigo and Love (2016) for panel data.

7 To observe the transformation, we can postmultiply (3) by the P matrix, which is lower triangular ${u}_{it}P{=\mathrm e}_{it}$. Then ${u}_{1it}={e}_{1it}$ and ${u}_{jit}={e}_{jit}-{p}_{1it}\mathrm{u}_{1it}, -{p}_{2it}\mathrm{u}_{2it}\dots , {-{p}_{j-1it}u}_{j-1it}$ when P is normalized with 1 in the diagonal.

8 We use Stata for estimations (Abrigo and Love, 2016). One standard deviation for the innovation shocks is calculated.

9 Given the cross-sectional homogeneity assumption, the IR effect is the same for all countries. However, we recalculate the IR functions only for the Latin American countries in the sample.

10 Using productivity log differences all moduli of the companion matrix are strictly less than one, indicating that the VAR system is stable. The lag selection was based on the proportion of variation explained by the PVAR model.

11 This is because the φ parameters are nonlinear for lags higher than one.

12 Results available upon request.

13 Given the sample size, we used target p-values of 0.001 (or less) for dummies and 0.01 for economic explanatory variables and country effects.

14 The long-run elasticities are derived from $\Delta y_{it} = \Delta k_{it} = \Delta x^{\prime}_{it} = 0$.

15 A useful approximation of the critical values of the *t-statistics* from the response function, which could be seen as a multivariate unit roots, is given by the rule “3–2–3”, that is the critical value is *-3–0.2 K–0.3 (d* − *1)* where *K* is the number of variables in the long run relationship and *d* is the number of deterministic components such as constant, step dummies and trends. This critical value can be applied to panel data models in the case of OLS estimates using data pooling with country dummy effects.

16 Both estimations were implemented in Stata.

17 Cluster-robust standard errors, calculated in Stata, are reported. Using these, results for significance and cointegration are similar to those when using ordinary least squares standard errors of standard errors adjusted by cross-dependence (Driscoll-Kraay standard errors obtained when using the *xtscc* command in Stata).

18 Initially the log difference of construction productivity was also significant; it became insignificant when instrumented with its own capital–labor ratio (lagged log level and log differences).

19 Insignificant differential effects were found for LAC countries when we test multiplicative dummies of the coefficients.

20 The long-run variance is calculated as J’V J, where V is the variance and covariance matrix of estimated coefficients (from Table 7) and J is the vector of first derivatives of the long-run parameters with respect to the estimated parameters. See Banerjee et al. (1993). For example, for the long-run elasticity with respect to *tsc* labor productivity (${~}^{{\beta }_{s,tsc}}\!\left/ \!\!{~}{{\delta }_{s}}\right.)$, we have J’ = (${~}^{1}\!\left/ \!\!{~}{{\delta }_{s}}\right., {~}^{ {\beta }_{s,tsc}}\!\left/ \!\!{~}{{\delta }_{s}^{2}}\right. )$.

21 In fact, this underestimates the full required effort in transport sector productivity, since the sector is experiencing indirect effects of the COVID-19 productivity shock in the wholesale, retail, and hospitality sector, as shown in the PVAR analysis of Sect. 3. The additional increase in the productivity of transport needed to compensate for these indirect effects can be approximated using the estimates reported in Table 4. That table showed that a negative shock of one standard deviation in wholesale, retail, and hospitality services provokes a loss in the productivity of transport of 2.1% for the whole sample and 3.1% for the LAC countries.
