# Supplementary material for: Sectoral Productivity Growth, COVID-19 Shocks, and Infrastructure
Source: Econ Disaster Clim Chang. 2022 Jan 21;6(1):1–28. doi: 10.1007/s41885-021-00098-z (PMC8777183; doi:10.1007/s41885-021-00098-z)
Supplement: Supplementary file 1 — Supplementary file1 (DOCX 255 kb) [file 41885_2021_98_MOESM1_ESM.docx]

## Online Appendix A. KLEMS Database

We compiled data available for KLEMS projects across countries, focusing on gross value added GVA), capital stock, employment, employees and TFP (*tfp_i*) as variables, all with sectoral disaggregation. From nine sectors (agriculture; mining; manufacturing; utilities; construction; trade and hospitality; transport, storage, and communications; finance, insurance, and real estate; public and social services) we aggregated granular data available for subsectors. For TFP (expressed as an index) we computed weighted averages across the corresponding sectors when necessary, weighting with sectoral GVA.

The complete dataset includes 26 countries spanning 20 years (1995-2015) that can be taken from the KLEMS projects. Eight countries are covered by the LAKLEMS project.^[[1]](#footnote-1)^ We also included 12 countries from the EUKLEMS^[[2]](#footnote-2)^ dataset; for the remaining 6 countries we used data from World KLEMS^[[3]](#footnote-3)^ or from strictly KLEMS-compatible projects drawing on official national accounts data. From these 6 countries we excluded India and Thailand upon inspection about the quality of data. Our final sample thus comprises 24 countries; sources are detailed in Table A.1. Figure A.1 outlines data availability across years for each country. Missing data were filled in with compatible national accounts data in the case of sectoral gross value added, capital stock, and employment. The main constraint in trying to take advantage of EUKLEMS’s sectoral granularity is the aggregation of LAKLEMS, which shapes the sectoral definition we use for this paper.

In order to make GVA and capital stock measures comparable across countries, we expressed all figures in 2010 dollars (purchasing power parity) using conversion factors from the World Bank.^[[4]](#footnote-4)^ Labor productivity (*y_i*) measures were expressed as *GVA/L*; capital stock per worker (*k_i*) as *K/L*. We calculated the percentage of workers who are salaried employees (*remun_i*) as a potential proxy for labor formality. These estimates span sectors, countries, and years.

**Table A.1.**

| **KLEMS Data Sources** | | |
| --- | --- | --- |
| *code* | *country* | *Source* |
| AT | Austria | EUKLEMS |
| CZ | Czech Republic |  |
| DE | Germany |  |
| DK | Denmark |  |
| ES | Spain |  |
| FI | Finland |  |
| FR | France |  |
| IT | Italy |  |
| NL | Netherlands |  |
| SE | Sweden |  |
| UK | United Kingdom |  |
| US | United States |  |
| CL | Chile | LAKLEMS |
| CO | Colombia |  |
| MX | Mexico |  |
| EL | El Salvador |  |
| CR | Costa Rica |  |
| HO | Honduras |  |
| PE | Peru |  |
| DO | Dominican Republic |  |
| CN | Canada | World KLEMS & Statistics Canada |
| JP | Japan | RIETI's Japan Industrial Productivity Database 2018 (JIP) |
| IN | India | Reserve Bank of India's KLEMS Database |
| AS | Australia | Australian Bureau of Statistics |
| KO | Korea | Bank of Korea & GGDC |
| TH | Thailand | National Economic & Social Development Council of Thailand & GGDC |

**Figure A.1.**

**
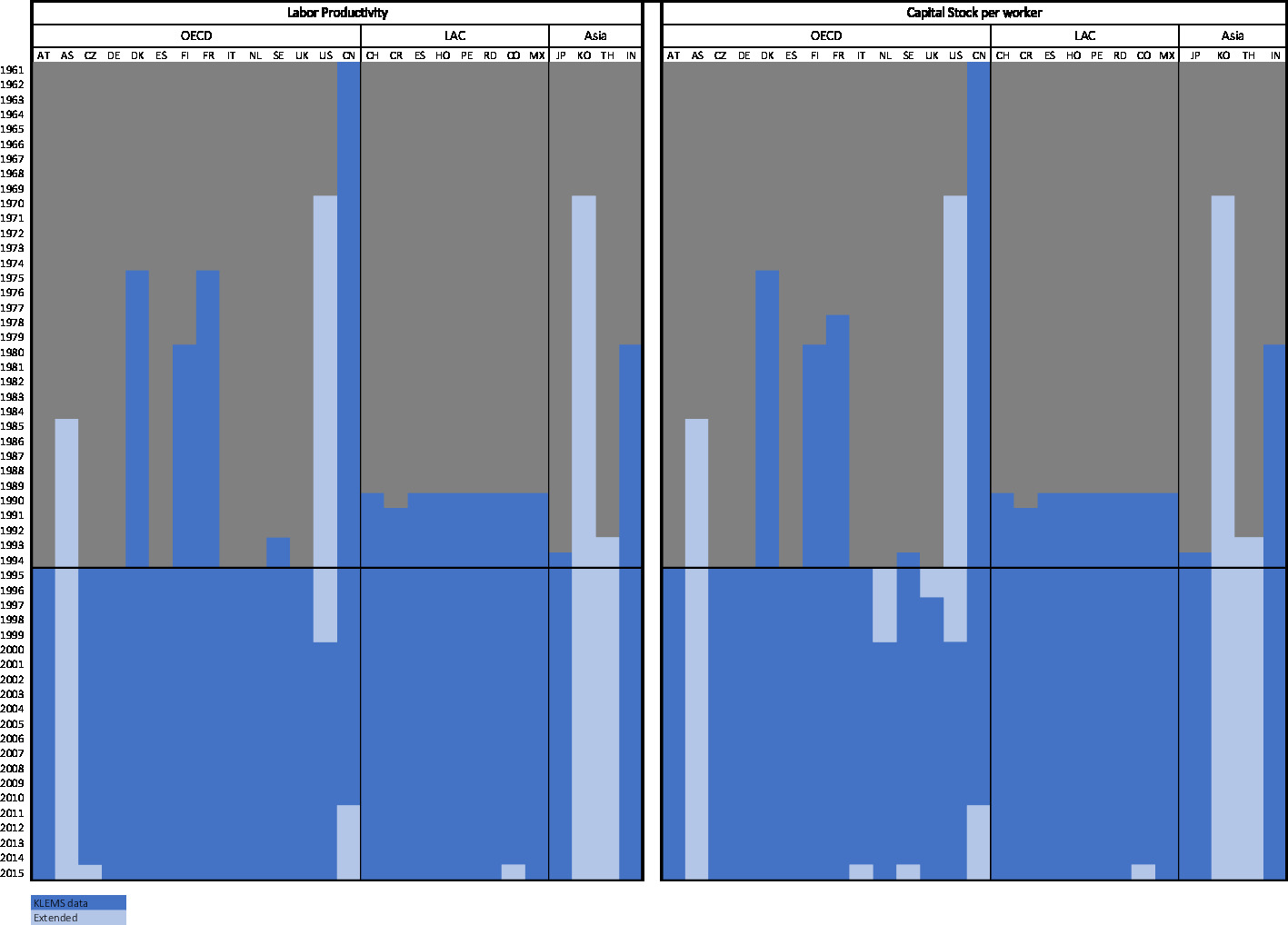
**

As control variables, we included a human capital index from Penn World Table 9.1,^[[5]](#footnote-5)^ and a measure of trade openness (exports plus imports as a percentage of GDP), using data from the World Bank. The complete set of variables is displayed in Table A.2.

**Table A.2.**

| **Variable Definition** | | | | |
| --- | --- | --- | --- | --- |
| *code* | *Variable description* | *Observations* | *Unit* | *Source* |
| **y_i** | **Labor productivity** |  | Thousand gross value added 2010 PPP dollars per person engaged | KLEMS |
| **k_i** | **Capital stock per worker** | All assets | Thousand gross value added 2010 PPP dollars per person engaged | KLEMS |
| **tfp_i** | **TFP Index** | TFP (value added based) | Index | KLEMS |
| **tfp2_i** | **TFP Index** | Calculated with average sample α_i_ | Log-thousand GVA 2010 dollars per person engaged | Own based on KLEMS |
| **va_i** | **Gross Value Added** |  | Thousand 2010 dollars | KLEMS |
| **e_i** | **Employment** |  | Thousand engaged | KLEMS |
| **remun_i** | **% of workers that are employees** |  | % of engaged workers | Own based on KLEMS |
| **hc** | **Human Capital Index** |  | Index | PWT 9.1 |
| **trade** | **Trade as % of GDP** | Exports+Imports | % of GDP | World Bank |

## Online Appendix B. High-Frequency Data Sources and Methodology

Data on monthly sectoral output was collected mainly from national statistical institutions for LAC countries and from OECD Stat^[[6]](#footnote-6)^ for OECD-Europe averages. Table B.1 lists the data availability and sources for each country.

**Table B.1.**

We studied four different monthly time series: aggregate GDP, construction, manufacturing, and wholesale and retail trade. In each case we seasonally adjusted monthly series (using Census X-12) and also expressed them as year-on-year growth rates whenever the corresponding national statistical office did not present the series in that format.

For each of the four categories we computed average year-on-year growth rates across countries. Not all LAC countries publish monthly data distinguishing retail from wholesale trade.

## Online Appendix C. Impulse-Response Figures for Sectoral Growth Rates

## of Labor Productivity (Table 3)

**Figure C.1. Orthogonalized Impulse-Response Functions for an Exogenous Shock**

**in Wholesale and Retail Trade and Hospitality, OECD and LAC**

Entire sample

LAC only

**Figure C.2. Orthogonalized Impulse-Response Functions**

**for an Exogenous Shock in Manufacturing, OECD and LAC**

Entire sample

LAC only

**Figure C.3. Orthogonalized Impulse-Response Functions**

**for an Exogenous Shock in Construction, OECD and LAC**

Entire sample

LAC only

## Online Appendix D. Coefficients of Impulse-Response Functions for Total Factor Productivity Shocks

| **Table A.3 Orthogonalized Impulse-Response Functions**  **for Sectoral Growth Rates in Total Factor Productivity** |
| --- |
|  |

1. <http://laklems.net/stats/result> [↑](#footnote-ref-1)
2. <https://euklems.eu/> [↑](#footnote-ref-2)
3. <http://www.worldklems.net/data.htm> [↑](#footnote-ref-3)
4. <https://data.worldbank.org/> [↑](#footnote-ref-4)
5. <https://www.rug.nl/ggdc/productivity/pwt/?lang=en> [↑](#footnote-ref-5)
6. <https://stats.oecd.org/> [↑](#footnote-ref-6)
